# Enhancing Executive Control: Attention to Balance, Breath, and the Speed *Versus* Accuracy Tradeoff

**DOI:** 10.3389/fpsyg.2020.00180

**Published:** 2020-03-06

**Authors:** Varsha Singh, Vaishali Mutreja

**Affiliations:** ^1^Department of Humanities and Social Sciences, Indian Institute of Technology, New Delhi, India; ^2^Toronto Western Hospital, Krembil Research Institute, Toronto, ON, Canada

**Keywords:** attention, executive control, yoga, posture, breath control, speed–accuracy

## Abstract

Malleability of executive control and its enhancement through yoga training is unclear. In Study 1, participants (yoga group; *n* = 27, mean = 23.27 years) were tested on executive control tasks pre- and post-8 weeks of yoga training. The training focused on attention to postural control during yoga asanas and respiratory control during pranayama-breathing (30 min each of postural and breath control training, biweekly). Yoga training was assessed *via* performance ratings as to how well a posture was executed and by examining errors that reflected inattention/failures in postural and breath control. We also explored whether attentional demands on motor and respiratory control were associated with three components of executive control (working memory, cognitive flexibility, and inhibition) during nine executive control tasks. Partial correlation results revealed that the three components of executive control might be differentially impacted by postural and breath control and selectively associated with either speed or accuracy (except for cognitive flexibility). Attentional demands influenced the link between postural, breath, and cognitive control. In Study 2, comparisons between a yoga group and a gender-matched control group (control group; *n* = 27, mean = 23.33 years) pointed toward higher working memory accuracy and a better speed–accuracy tradeoff in inhibitory control in the yoga group. A ceiling-practice effect was addressed by examining yoga practice learning (i.e., practice-induced change in postural and breath control reflected in ratings and errors) on executive control performance across two sets of tasks: repeatedly tested (pre- and post-8 weeks) and non-repeatedly tested (post-8 weeks). Attention to motor and respiratory control during yoga might be considered as a potential mechanism through which specific components of executive control in young adults might be enhanced potentially *via* altering of speed–accuracy tradeoff.

## Introduction

Malleability within different components of executive control in early adulthood is not well understood (e.g., Diamond and Lee, 2011; Diamond and Ling, 2016; Friedman et al., 2016). Even though attention and executive control processes could be influenced by yoga and other mindfulness practices (Jha et al., 2007; Teper and Inzlicht, 2013), the mechanism through which such practices enhance attention and executive control remains unknown. Scholars have pointed out several limitations of studies examining the cognitive enhancement of yoga and mindfulness practices, namely, a lack of specificity in defining the construct underlying the practice, lack of precision with measuring the construct, and failure to establish a link between the construct and the cognitive function presumably enhanced (Davidson and Dahl, 2018; Van Dam et al., 2018). The practice of yoga comprises postures (asanas) and breathing (pranayama) (Woodyard, 2011; Sengupta, 2012). Furthermore, attention training enhances different components of executive control by regulating the speed-accuracy tradeoff. The three components of executive control of greatest interest to the present study are working memory (i.e., storing information in the mind long enough to use it), cognitive flexibility (i.e., changing perspectives by shifting attention), and inhibition (i.e., selectively attending to one stimulus while ignoring another) (Miyake et al., 2000; Diamond, 2013).

Performing yoga postures involves planned movements and attaining specific body poses while maintaining balance. Attention to breathing involves monitoring, anticipating, and controlling the rate of respiration (e.g., slow *vs.* fast). In other words, both practices involve attention to two autonomic processes: motor and respiratory control. Studies on attention and motor control indicate that when attention is focused away from the body, natural control of body movement is enhanced (McNevin et al., 2003). Conversely, cognitive tasks that deplete attention interfere with postural balance (Balasubramaniam and Wing, 2002). Similarly, pranayama requires attending to the autonomic process of breathing (focusing on the breath) and though attention depletion due to cognitive load alters breathing (Grassmann et al., 2016), such depletion likely resets the autonomic nervous system (Jerath et al., 2006). It is possible that the attentional demands required for bringing these ordinary autonomic processes under volitional control transform these into controlled and goal-directed activity by using cognitive resources such as working memory, planning-flexibility, and inhibition. Therefore this could be one mechanism through which yoga practice enhances executive control.

There could be a differential role of attention during posture and breath control practice, and these two components of yoga training likely have separable influences on executive control. Though asanas and pranayama both aim at controlling autonomic processes, others have recommended that the effects of breath control be examined separately from those of postures (Jerath et al., 2006; Trakroo et al., 2013). During posture training, the eyes are open in order to regulate movements by imitating an external referent (e.g., yoga instructor, a picture, or a video), whereas breath control exercises are performed with the eyes closed, cultivating internal awareness by curtailing external referents. Attentional demands during monitoring and retaining an external referent will be different from the demands of maintaining self-focus while inhibiting external referents. Furthermore, the two types of yoga training differentially involve working memory and distinctly regulate speed of processing (e.g., executing a yoga posture involves motor planning and requires working memory; Anguera et al., 2011; Seidler et al., 2012). For instance, executing a posture relies on convenient access to a mental image of a body arrangement in the form of a specific posture (e.g., visuospatial image of a posture). Conversely, breathing exercises have no such memory demand, as no external reference or visual image is necessary to focus, monitor, or regulate breathing. Additionally, the distinction between postural control and breath control practice will involve the two components of working memory (visuospatial and verbal) differently. Verbal working memory, known as the “phonological loop,” stores verbal or articulatory information, whereas a “visuospatial sketchpad” serves as the storage for non-verbal spatial information (Baddeley and Hitch, 1974). Postural control disrupts visuospatial rather than verbal working memory (Gunduz Can et al., 2017); whereas breath control is known to selectively affect visuospatial working memory (Jella and Shannahoff-Khalsa, 1991; Telles et al., 2012). This is mainly because the respiratory system plays a critical role in speech and articulation (Ackermann and Riecker, 2010). Next, attention toward slowing paced movements improves postural control (Wu, 2002), and slow-paced breathing has the most evident cognitive benefits (Pal and Madanmohan, 2004; Jerath et al., 2006). However, some have found that both slow- and fast-paced breathing enhances cognitive control (Sharma et al., 2014). Slow movements during standing yoga postures require maintaining postural control against gravity; postural control failures pose a risk of losing body balance and falling. However, regulating the speed of breathing (fast or slow-paced) typically occurs in a sitting position, posing minimal or no risk of loss of balance and subsequent falls. Furthermore, respiration contributes to the rhythm or speed of brain functions (Heck et al., 2017); therefore, attentional demands during speed-regulation of breath control will differ from those of posture control, revealing differential associations with speed of processing during executive control.

The role of attention in linking motor, respiratory, and cognitive control can be delineated by demonstrating that variations in attentional demands during posture and breathing exercises are interlinked with executive control. Some postures, breathing exercises, and executive control components are less demanding than others; thus, attention is the key link between the three control systems. In this regard, the goal of Study 1 was to examine how the two components of yoga training (posture and breath control) are associated with three distinct components of cognitive control. In Study 2, we compared a yoga training group with an age–gender–education matched control group to examine changes in cognitive control as a function of yoga training.

## Study 1

This study investigated whether two control systems (motor and respiratory control) involved with yoga postures (asanas) and breathing (pranayama) are differentially linked with speed and accuracy when performing three types of executive control tasks. Furthermore, the present study examined whether attentional demands alter the relationship between motor, respiratory, and executive control. This also involved testing the association between changes in motor and respiratory control through yoga training and executive control. We hypothesized that the three executive control components (speed and accuracy) would respond differently to the two types of yoga practice and that attention would accentuate the link between motor, respiratory, and cognitive control. In short, attention would be the mechanism through which the two yoga practice components would enhance executive control.

### Materials and Methods

#### Measures

Nine tasks from the psychological experiment builder language (PEBL) were used to assess working memory, cognitive flexibility, and inhibition (Piper et al., 2016). Performance under high attentional demands was assessed by analyzing task complexity within each of the nine tasks (i.e., performance on task trials that were difficult/harder had higher demands as compared to performance on task trials that were simpler/easier).

##### Working memory

###### Digit span task (forward)

This task assessed reaction time (RT) and accuracy of verbal/phonological working memory, with the participant recalling digits (1–9) presented in an increasing order.

###### Corsi block test (forward)

This task assessed RT and accuracy of visuospatial working memory, which required participants recalling a sequence of blocks presented in increasing order.

###### Mental rotation task

This task assessed RT and accuracy of visuospatial working memory (Berteau-Pavy et al., 2011) and involved deciding whether two-dimensional shapes presented side-by-side on the screen were the same or different when rotated clockwise or counterclockwise at 50, 100, and 200°.

During the Corsi block test and digit span task, working memory demands increased sequentially: blocks and digits of a longer length placed more attentional demand on the participant.

##### Planning and cognitive flexibility

###### Toward of Hanoi

This task assesses planning, problem solving, and flexibility while revising plans, as it comprises rule-based transferring of three disks from one peg to another goal peg.

###### Tower of London

This task assesses planning and flexibility as participants are required to move three colored disks of the same size with a goal of preparing a specified stack/disk arrangement.

###### Berg’s card sorting task

This task, modeled after the Wisconsin Card Sorting Task, measures rule learning and set shifting wherein a participant learns to sort stimuli based on three rule-changes (e.g., color, size, and form).

Although both the Tower of Hanoi and Tower of London are disk-transfer tasks used to assess planning and problem solving, the Tower of London is less demanding on working memory (Humes et al., 1997); however, for both tasks, attention and working memory load increases as the number of requisite steps increases (Spitz et al., 1984). Preservative errors are of greatest import during Berg’s card sorting task, as these errors are the result of failures to shift attention from an old rule to a new rule (Barcelo, 1999).

##### Inhibition

###### Simon task

This task has a stimulus (a colored circle) presented on the right or left side of the screen, and the goal of the task is to respond to the color of the circle by pressing a button (red = left shift and blue = right shift). During incongruent trials, a red circle appears on the right side and vice versa, influencing RTs and accuracy, referred to as the Simon effect. Inhibition is required to suppress a target location-based response.

###### Stroop task

The names of the four colors (e.g., “blue,” “green,” “red,” or “yellow”) appear one-by-one on a screen, and a keypad response is mapped to each color. The color of the word either matches the name of the color (congruent) or is a mismatch (incongruent). RTs are slower when the color of the ink mismatches the name of the color. The task assesses the inhibition of word reading during color naming.

#### Iowa Gambling Task (IGT)

This task assesses decision-making where the participant has to choose between short-term risky *vs.* long-term safe rewards. A deficit reflects impulsivity and failure to inhibit the choice of an immediate, but risky, reward option.

In both the Simon and Stroop task, incongruent trials are more demanding than congruent trials. In the IGT, attentional and working memory demands during initial trials (blocks two and three) are higher (Bagneux et al., 2013).

##### Mood measure

The Positive and Negative Affect Schedule (PANAS) was used to assess mood. The mood measure was used for assessing the postsession mood (i.e., mood immediately after completing a session) and its alteration over the period of training (i.e., changes in postsession mood from the start to the end of the 8-week training period). We considered the difference between the first two sessions and the last two sessions to reflect alteration in postyoga mood analyzed over the period of yoga training (8 weeks). Due to multiple PANAS measures for each participant and unequal number of mood measures between the participants, Cronbach Alpha was not calculated.

##### Body Mass Index

BMI was calculated using the National Institute of Health protocol by dividing a participant’s weight in kilograms by the square of his/her height in meters.

#### Participants

Twenty-seven healthy young adults (mean age: 23.37, *SD*: 3.89; 17 men) volunteered for the study. Inclusion criteria were as follows: >18 years and willing to undergo yoga training (postural and breath control). Participants were recruited by a female research assistant (RA) by sending emails to the hostel and institute email groups, requesting for participation (call for participation). Fifty two participants responded to the call, of which, total twenty five participants were excluded; reasons for exclusion were as follows: participants gave baseline but did not come for yoga stating due to lack of time (12), attended less than half of yoga sessions (seven), and participants did not give retest (six: four had left the campus, two were unresponsive). The participants were assessed for known psychiatric illnesses by using the Mini International Neuropsychiatric Interview (MINI). All participants were undergraduate (59%) or postgraduate students (∼40%) and yoga-naïve (self-declared first-time receivers of any form of yoga training). The ethics committee of the institute approved this study. All participants provided signed informed consent prior to participating. The participants also received a cash reimbursement (INR 500).

#### Procedure

After obtaining informed consent and demographic information from the participants, four cognitive tasks were administered prior to the start of the yoga training (task order: digit span task, Tower of Hanoi, Simon task, and IGT). Postural and breath control training (i.e., asanas and pranayama sessions) was imparted as per a preset schedule (see Table 1). Researchers have expressed concern over the absence of a detailed protocol for replicability with mindfulness-related practice studies (Van Dam et al., 2018). Thus, a detailed protocol is presented (Table 2). At the end of every yoga session, participants completed the PANAS questionnaire. After the last training session, participants were contacted and were asked to come back after 5 days for a retest. Participants were retested on the four executive control tasks, and after a 10-min break, participants were administered five new cognitive tasks (task order: Corsi block test, mental rotation task, Stroop task, Tower of London, and Berg’s card sorting task). Data from the observation sheets of two research assistants, task output files, and PANAS sheets were entered into excel files and imported into a Statistical Software for Social Sciences (SPSS), version 18, database.

**TABLE 1:** Yoga posture and pranayama breathing protocol performed by the yoga group (*n* = 27).

**Postures**	**Duration**
Warm-up (on spot jogging)	5 min
Padahastasana	2 min
Virabhadrasana*	2 min
Trikonasana*	2 min
Katichakrasana	2min
Tadasana	2 min
Ardha Chakrasana	2 min
Pranamasana*	2 min
Vrikasana*	2 min
Break	5 min
Breathing	Duration
Abdominal breathing	5 min
Thoracic breathing	5 min
Brahma mudra	5 min
Alternate nostril breathing	5 min
Observing breath	5 min
Mood measure (PANAS)	5 min

*Bilaterally done postures (1 min per side).

**TABLE 2:** Training session details as suggested by Van Dam et al. (2018) for yoga group (*n* = 27).

Teacher information	Number/types of retreats attended	5
	Experience in contemplative instruction	10 years
	Formal contemplative training	10 years
	Formal clinical qualification	None
	Blinded to experimental hypotheses	Yes
Practice	Setting(s)	Student activity Centre
information	Physical	Open space/Large hall (as per the weather)
	Social	19–22 students
	Overall duration	8 weeks
	Frequency of meetings	Twice a week
	Average length of meetings	70 min
	Types of formal practice	Yoga and breath control
	Approximate total percentage of each type of practice	50% yoga posture and 50% breathing (see **Table 1**)
	Types of informal practice	None
	Logs, practice review, guided	Logs maintained for each guided session
	Types of instructional materials used	Verbal instructions and demonstration from the trainer
General	Instructor adherence assessed	Yes
information	Control group used	No
	Randomization/allocation method	No
	Adverse events monitored	Yes
Participant information	Inclusion criteria	Yoga-naïve, age: 18–30 years
	Exclusion criteria	Exclusion: Psychiatric illness (MINI)
	Prior meditation experience	None
Conflict	Formal: Funding agency	Faculty Interdisciplinary Research Project
	Informal: Financial benefit	None

#### Yoga Training Session

A certified and experienced (>10 years) yoga instructor (female, age: 42 years) performed yoga postures (30 min) and five breathing exercises (30 min). Two research assistants (one male and one female) recorded and rated participants’ performance during the posture and breathing sessions by using an observation sheet (see Appendix 1). To observe participants’ performance, the two research assistants were seated in a designated position that provided a clear view of the participants. The participants were equally divided between the two research assistants for observation (average session attendance = 12 participants). The participants and research assistants switched sides every session to ensure that both research assistants contributed equally to participants’ posture and breathing training ratings. The observation sheet was used to rate postures on a scale from 0 to 4 (0 denoting poor performance as compared to the instructor and 4 denoting precise performance and an exact replica of the instructor). Failure to maintain posture or balance and movements that were not a part of designated posture-related movements were counted as motor control failures or errors. Similarly, breathing sessions were rated on a scale from 0 to 4 (0 denoting poor performance and 4 denoting precise performance). All postures were performed in a vertical, standing position. The breathing exercises were performed in a sitting position. Errors were counted as a disruption in the specified breathing activity (e.g., opening eyes midsession, moving when asked to hold still, or failure to follow any other breathing instructions). Ratings reflected participants’ execution of the postural and breath control exercise compared to that demonstrated by the yoga instructor. To identify attention demands in posture and breath control, we classified postures and breathing exercises on the basis of the difficulty involved in execution. Classification of postures and breath control exercise on the basis of difficulty in execution (i.e., high *vs.* low demanding posture and breath control) rather than that of the participants on the basis of their ability to execute the posture or breath control (high/good *vs.* low/poor executers of postures and breath control exercises) enabled us to maximize the number of observations.

#### Variables and Data Analyses

Postural and breath control ratings as well as postural and breath control errors were treated as continuous variables (see observation sheet in Appendix 1). Accuracy and RTs were calculated to measure executive control performance on each task. Ratings and errors were negatively correlated for postures (*r* = −0.76, *p* > 0.01) and breathing (*r* = −0.71, *p* > 0.01); high motor and breath control were associated with fewer errors. Postures with a rating that is higher than the mean would be less demanding whereas postures with a low rating would be considered more demanding (i.e., difficult). Similarly, cognitive task trials with greater challenge were considered highly demanding trials. Partial correlations were analyzed to control for age, sex, and BMI. A first set of correlations tested the link between postural control (ratings and errors), breath control (ratings and errors), and cognitive task performance (accuracy/performance and RT) for (a) working memory (digit span task, Corsi block test, and mental rotation task), (b) planning and cognitive flexibility (Tower of Hanoi, Tower of London, and Berg’s card sorting task), and (c) inhibition (Simon task, Stroop task, and the IGT). A second set of correlations then tested the link between postural, breath control, and executive control by accounting for various attentional demands. The third set of correlation analyses addressed ceiling-practice effects (i.e., cognitive task improvement in accuracy and RTs due to practice and repeated task exposure). The link between motor, respiratory, and cognitive control learning was tested for repeated (pre and posttraining assessment) and non-repeated tasks (posttraining assessment). The average of pre and posttraining performance was used for the repeated tasks.

### Results and Discussion

Participant characteristics are listed in Table 3. Means and standard deviations for speed and accuracy observed across the nine executive control tasks are shown in Table 4.

**TABLE 3:** Sample characteristics (*N* = 27).

**Characteristic**	**Mean (*SD*), percentage**
Age	23.37 (3.89)
Body mass index	22.54 (2.57)
Sex	Male: 63%; Female: 37%
Handedness	RH: 100%; LH: 0%
Vision	Corrected: 63%
Education	UG: 59%; PG: 41%

**TABLE 4:** Descriptive table of speed and accuracy in general and highly demanding trials of the tasks representing the three components of executive control in the yoga group (*n* = 27).

**EF component**	**EF tasks**	**Task speed (RT)**	**Task accuracy (score)**	**Highly demanding trial speed (RT)**	**Highly demanding trial accuracy (score)**
Working memory	DS	73.3	101.34	18.85	–2.75
		(18.14)	(33.44)	(10.76)	(1.21)
	Corsi	69.22	71.19	18.41	–3.86
		(25.71)	(31.00)	(15.38)	(1.44)
	MRT	425.33	94.63	59.87	–6.71
		(192.2)	(25.83)	(64.41)	(23.12)
Cognitive flexibility	ToH	180.84	17.54	61.2	7.54
		(67.58)	(12.92)	(67.38)	(10.02)
	ToL	682.02	16.89	–94.89	–6.45
		(170.05)	(6.23)	(92.65)	(3.36)
	BSCT	218.89	27.04	30.96	62.52
		(52.13)	(13.04)	(22.96)	(19.19)
Inhibition	Simon	60.4	125.36	1.53	–1.43
		(17.22)	(29.88)	(1.96)	(1.38)
	Stroop	118.91	134.41	2.51	–0.82
		(20.48)	(13.4)	(2.56)	(2.06)
	IGT	127.83	13.67	3.74	–3.04
		(58.77)	(21.83)	(18.02)	(12.72)
					

#### Working Memory

The association between postural and breath control and working memory was assessed using the digit span task (forward), Corsi block task (forward), and mental rotation task. Performance ratings and errors across the posture and breathing exercises were analyzed in comparison to speed and accuracy within the three working memory tasks (Table 5). Errors during breath control were associated with high accuracy on the digit span task (*r* = 0.42; *p* = 0.04). After factoring in attentional demands (Table 6), errors during the less demanding breath control exercises were associated with higher accuracy on the more demanding digit span trials (longer span; *r* = 0.45; *p* = 0.03). Postural control ratings during the less demanding postures were associated with faster RTs on the more challenging Corsi block trials (trials with longer block spans; *r* = −0.42; *p* = 0.04). Inattention during the less demanding breath control exercises was associated with slower RTs on the more demanding mental rotation task trials (mirror image; *r* = 0.40; *p* = 0.05). Overall, these results suggest a possible link between the demands on motor and respiratory control and the attentional demands on working memory.

**TABLE 5:** Postural, breath control, mood, and cognitive control tasks (accuracy and RT) in the yoga group (*n* = 27).

**Task Performance**	**Posture-control (motor control)**		**Breathe-control (respiratory control)**		**Mood (PANAS)**	
			
**(cognitive-control)**						
	**Posture rating**	**Posture error**	**Breathing rating**	**Breathing error**	**Positive**	**Negative**
**Working Memory**						
DS score	–0.02	0.10	–0.27	0.42*	–0.11	0.03
DS RT	0.18	–0.31	0.06	–0.23	–0.02	0.30
Corsi score	–0.26	0.11	0.10	0.08	0.06	0.10
Corsi RT	–0.38	0.10	–0.18	0.24	–0.12	0.32
MRT score	–0.21	0.06	0.14	–0.00	–0.06	–0.20
MRT RT	–0.12	0.10	–0.06	0.17	–0.00	–0.26
**Planning—cognitive flexibility**						
ToH score-*R*	0.19	–0.11	0.05	–0.04	0.05	0.04
ToH RT-*R*	0.37	–0.09	0.43*	−0.48*	0.27	–0.01
ToL score	–0.26	0.14	0.19	0.20	–0.02	0.01
ToL RT	−0.46*	0.40*	–0.12	0.38	–0.04	0.15
BCST score	0.36	–0.19	–0.03	0.11	–0.04	–0.02
BCST RT	0.24	–0.22	−0.40*	0.40*	–0.06	–0.03
**Inhibition**						
Simon score-*R*	0.13	–0.32	–0.36	0.12	–0.19	–0.02
Simon RT-*R*	0.29	–0.39	–0.13	–0.00	–0.07	0.22
Stroop score	0.11	–0.17	0.13	0.12	–0.02	0.28
Stroop RT	0.07	0.09	–0.15	–0.07	0.26	–0.07
IGT score-*R*	0.06	–0.08	0.28	0.02	0.07	0.23
IGT RT-*R*	–0.17	0.24	0.09	–0.06	0.06	0.05

*Correlation significant at 0.05 level (two-tailed). DS score = block span × correct score; DS RT = RT for items; Corsi score = block span × correct score; Corsi RT = RT for items. MRT = Mental Rotation Task correct score/correct responses; MRT RT (Mental Rotation Task RT) = Task RT; ToH score = Tower of Hanoi Task score is steps minus shortest; ToH RT = Task RT; ToL score = Tower of London correct score/correct responses; ToL RT (Tower of London RT) = Task RT; BCST score = Berg’s Card Sorting Task total error is total errors (preservation + non-preservation); BCST RT (Berg’s Card Sorting Task RT) = Task RT. Simon score = correct responses; Simon RT = Task RT; Stroop score = Interference correct score; Stroop RT = Task RT; IGT score = Iowa Gambling Task net score [(C + D) − (A + B)]; IGT RT = Task RT.

**TABLE 6:** Postural, breath control, mood, and high demanding trials of cognitive control tasks (accuracy and RT) in the yoga group (*n* = 27).

**Task performance**	**Posture-control demand**				**Breath control demand**				**Mood (PANAS)**	
			
**(High cognitive-control)**										
	**Low-R**	**High-R**	**Low-E**	**High-E**	**Low-R**	**High-R**	**Low-E**	**High-E**	**PA**	**NA**
**Working memory**										
H-DS score	–0.14	–0.07	0.32	0.13	–0.33	–0.26	0.45*	0.36	–0.12	–0.06
H-DS RT	–0.05	0.00	–0.11	–0.19	–0.15	–0.30	0.13	0.07	–0.08	–0.22
H-Corsi score	–0.05	0.24	–0.06	–0.24	0.15	0.02	0.08	0.10	–0.29	–0.01
H-Corsi RT	−0.42*	–0.01	0.07	–0.04	–0.24	–0.14	0.29	0.25	–0.19	0.31
H-MRT score	0.01	–0.25	0.20	0.03	0.19	–0.31	–0.03	0.33	–0.20	–0.13
H-MRT RT	–0.11	–0.00	0.10	–0.13	–0.14	0.13	0.40*	0.14	–0.28	0.03
**Planning—cognitive flexibility**										
H-ToH score	–0.12	–0.25	0.01	0.29	–0.02	–0.16	0.07	0.21	0.03	0.03
H-ToH RT	0.08	0.15	–0.14	0.02	–0.06	0.06	0.07	0.01	0.13	0.22
H-ToL score	0.22	0.21	−0.40*	–0.17	0.10	–0.10	–0.12	0.13	0.02	0.15
H-ToL RT	0.10	0.03	0.01	0.21	0.04	–0.25	–0.00	0.09	0.08	0.22
H-BCST score	–0.34	−0.58**	0.44*	0.59**	–0.10	–0.25	0.01	0.37	0.15	0.02
H-BCST RT	0.30	0.56**	–0.38	−0.48*	0.05	0.06	0.08	–0.09	–0.09	0.03
**Inhibition**										
H-Simon score	0.10	–0.01	0.06	0.22	0.01	0.22	–0.04	–0.14	0.24	–0.26
H-Simon RT	0.25	0.05	–0.10	–0.03	0.31	0.46*	–0.17	–0.36	0.08	–0.00
H-Stroop score	0.13	–0.05	–0.16	0.00	0.11	–0.25	0.08	0.38	0.08	0.24
H-Stoop RT	0.00	0.10	–0.08	–0.09	–0.22	–0.09	–0.09	–0.20	0.02	–0.03
H-IGT score	–0.05	–0.08	0.17	0.17	0.09	0.30	–0.09	–0.27	0.08	–0.02
H-IGT RT	0.01	–0.13	–0.04	0.12	0.14	–0.18	–0.27	0.02	0.24	0.12

**Correlation is significant at 0.01 level (two-tailed); *Correlation is significant at 0.05 level (two-tailed). H-DS Score = block 2 (difficult) correct score - block 1 correct score (easy), and H-DS RT = RT of block 2 - RT of block 1; H-Corsi Score = scores of difficult block (block2) − scores of easy block (block1); H Corsi RT = RT of block 2 – RT of block 1; H MRT Score: accuracy of reverse object or Cond1 (difficult) - accuracy of original object image or Cond0 (easy); H MRT RT = RT for reversed object image − RT of original object image; H-ToH Score = correct score of trials with higher no. of steps (difficult) - correct score of trials with lower no. of steps (easy); H-ToH RT = RT of difficult – RT of easy trials; H-ToL Score = scores of difficult blocks (block 3&4; length 6&7) - scores of easy block (block 1&2; length 4&5); H ToL RT = RT of difficult block - RT of easy block; H BCST Score: percentage of preservation errors; H BCST RT: RT of total errors - RT of preservation errors; H-Simon Score = Interference suppression accuracy is incongruent trial’s correct score (difficult) - congruent trial’s correct score (easy); H-Simon RT = RT of incongruent - RT of congruent trials; H-Stroop Score = Incongruent trial’s correct score (difficult) - congruent trial’s correct score (easy); H-Stroop RT = RT of incongruent - RT of congruent trials; H-IGT score = net score of blocks 2&3 (difficult) - net score of blocks 4&5 (easy); H-IGT RT = RT of difficult blocks - RT of easy blocks.

We speculate that posture and breath control might have had a selective effect on visuospatial *vs.* verbal working memory. Responsiveness in regard to spatial or object rotation (mental rotation task) in reference to breath control is aligned with findings that breathing-related training improves spatial memory accuracy among women when compared with men (Jella and Shannahoff-Khalsa, 1993). Postural control seemed unrelated to verbal working memory as assessed by the digit span task. Others have also found that non-posture-related training, as compared to karate training (motor control training), has no effect on verbal working memory (Jansen et al., 2017). Non-posture-related training combined with goal training for substance abuse also reveals no improvement to verbal working memory among a trained group (Alfonso et al., 2011), or there is a weak link between posture control and verbal working memory (Telles et al., 2007, 2008). However, studies using a combined analysis of postures and breathing make it difficult to delineate training-induced improvement in verbal working memory (e.g., Purohit and Pradhan, 2017). Results might be suggestive of a selective link between pranayama breathing and verbal working memory possibly because verbal working memory (digit span task) implicates the phonological loop (Wang and Bollugi, 1994; Christie et al., 2013) and is associated with the respiratory process of breath control (Lau et al., 2015). Therefore, the two components of working memory might have responded differently to the two yoga training components.

#### Planning and Cognitive Flexibility

Three tasks were used to assess the association between postural and breath control and planning and cognitive flexibility: Tower of Hanoi, Tower of London, and Berg’s card sorting task. Breath control was correlated with RTs: better breath control was associated with slower RTs (*r* = 0.43; *p* = 0.03), while lower breath control was associated with faster RTs on the Tower of Hanoi task (*r* = −0.48; *p* = 0.02). Given that breath awareness is a measure of present moment awareness (Levinson et al., 2014), attention to the present moment facilitates insight during planning (Ostafin and Kassman, 2012). Mindfulness-related training improves planning and RTs among 10–13-year-old girls (Manjunath and Telles, 2001), adolescent girls with ADHD (Kiani et al., 2016), and patients with frontal lobe damage show better planning after breathing-focused mindfulness (Levine et al., 2011). Better postural control was also associated with faster RTs (*r* = −0.46; *p* = 0.03), whereas worse postural control was linked with slower RTs on the Tower of London task (*r* = 0.40; *p* = 0.05). Less demanding postures were related to lower accuracy on the demanding Tower of London trials (*r* = −0.40; *p* = 0.05).

Cognitive flexibility is reflected in preservative errors on the Berg’s card sorting task, as these errors occur due to a failure to shift attention to a new sorting rule (Barcelo, 1999). Results suggested that better breath control was associated with faster RTs (*r* = −0.40; *p* = 0.05), while worse breath control was linked with slower RTs (*r* = 0.40; *p* = 0.05). Breath control seems to be associated with the regulation of speed/reaction time when shifting attention. When factoring in attentional demands, the less demanding postures were associated with more preservative errors (*r* = 0.44; *p* = 0.03). Conversely, better performance on the high-demanding yoga postures was related to fewer preservative errors (*r* = −0.58; *p* = 0.003) but slower RTs (*r* = 0.56; *p* = 0.004). Errors during the challenging postures were linked with faster RTs (*r* = −0.48; *p* = 0.02). Performing high demanding yoga postures with precise motor control indicates greater cognitive flexibility (fewer preservative errors); however, independent of demand, unplanned movements during yoga postures (i.e., posture errors) were associated with attention shifting failures. Attention to a goal-directed movement needed for performing a yoga posture seemed to be associated with attention shifting when learning a new rule, whereas breath control might be linked with the speed of planning and flexibility. Breath-focused mindfulness (MBSR) did not affect cognitive flexibility among fifth-grade children (Wimmer et al., 2016) or patients with multiple sclerosis (Amiri et al., 2016), suggestive of the importance of postural control training. However, more focused efforts are needed to delineate the responsiveness of this cognitive domain to motor and respiratory components of the training and to test whether the interaction of motor and breath control impacts the speed–accuracy tradeoff.

#### Inhibition

Associations between inhibition and postural and breath control training were assessed with the Simon task, Stroop task (Color), and IGT. Postural and breath control were not associated with Stroop task performance. Other researchers have also found no effects of breath-focused training on Stroop task performance (Semple, 2010; Lee and Orsillo, 2014), citing ceiling effects (Anderson et al., 2007; Moore et al., 2012). Breath control was associated with Simon task performance but only when attention demands were considered: errors during high demand breath control exercises were associated with slower RTs (*r* = 0.46; *p* = 0.02). No other correlations were significant. Incongruence between the stimulus and response location produces slower RTs, reflecting the Simon Effect (Scerrati et al., 2017). Compared to the Stroop task, the Simon task is less verbal (Stroop task requires suppressing the conflict between naming a color *vs.* a word; Scerrati et al., 2017) and possibly relies more on spatial processing. A speculation that needs rigorous examination in future might be the role of working memory (i.e., spatial *vs.* verbal) in yoga-based enhancement of inhibitory control.

Counterintuitively, inhibiting impulsive choices in the IGT was not linked with postural and breath control. The IGT performance depends on somatic information that conveys body-states to the brain (Brinkmann, 2006), and inhibitory control is dependent on working memory and executive functions (Gansler et al., 2011; Bagneux et al., 2013). However, tertiary education and explicit knowledge interferes with somatic-guided decision-making (Evans et al., 2004). Others have observed that somatic awareness or attention to somato-sensory processes did not improve inhibition during longer-term decision-making (Cui et al., 2015); however, it is possible that the effects of somatic awareness and attention training are evident in a longer term and remain relatively implicit. Results are suggestive of inhibition being one of the most challenging domains for assessing cognitive-enhancement in younger adults.

#### Mood, Posture, and Breath Control

Mood assessments were taken immediately after the training session to reflect the most immediate training-altered affect. Posttraining mood showed no associations with any of the tasks. However, changes in posttraining mood (difference in mood ratings between the initial and last sessions), specifically negative mood was associated with diminished inhibitory control (Simon task) and faster RTs (IGT) (*r* = −0.43; *p* = 0.04). Posture, breathing, and relaxation training has been shown to increase positive mood and decrease negative mood (Narasimhan et al., 2011), whereas others observed that breath-focused mindfulness training tends to have less of an effect on mood (Eisenbeck et al., 2018). Posttraining mood might have contributed to the cognitive benefits of yoga training, possibly a negative posttraining mood being associated with fewer cognitive benefits. More efforts are needed to identify the effect of immediate mood or mood alterations on cognitive enhancement accrued from yoga training.

#### Yoga-Learning and Ceiling-Practice Effects

Executive control task performance tends to improve when tested twice, as is the case with a pre and postintervention design. This indicates a practice effect whereby a ceiling effect suggests that such improvement among healthy participants has a threshold or ceiling (Moore et al., 2012). To address a possible ceiling effect in the present pre and postyoga training comparison, we analyzed whether the difference in posture and breath control over the period of yoga training and accompanying mood changes (difference between the first and last two sessions) were correlated with two blocks of executive control tasks: (a) repeated tasks, wherein differences in pre and postintervention task performance was analyzed (i.e., four tasks that were repeated after the yoga training) and (b) non-repeated tasks, wherein task performance was assessed only once after the yoga training (five non-repeated tasks) (Table 7). This enabled a comparison as to differences in executive control performance as a function of a practiced *vs.* non-practiced task. It was expected that a celling effect would be more likely on the repeated/practiced tasks.

**TABLE 7:** Correlation of learning-induced changes in postural, breath control with mood, and ceiling-practice effect (retested and non-retested) in cognitive control task (accuracy and RT) with attention demands (yoga group: *n* = 27).

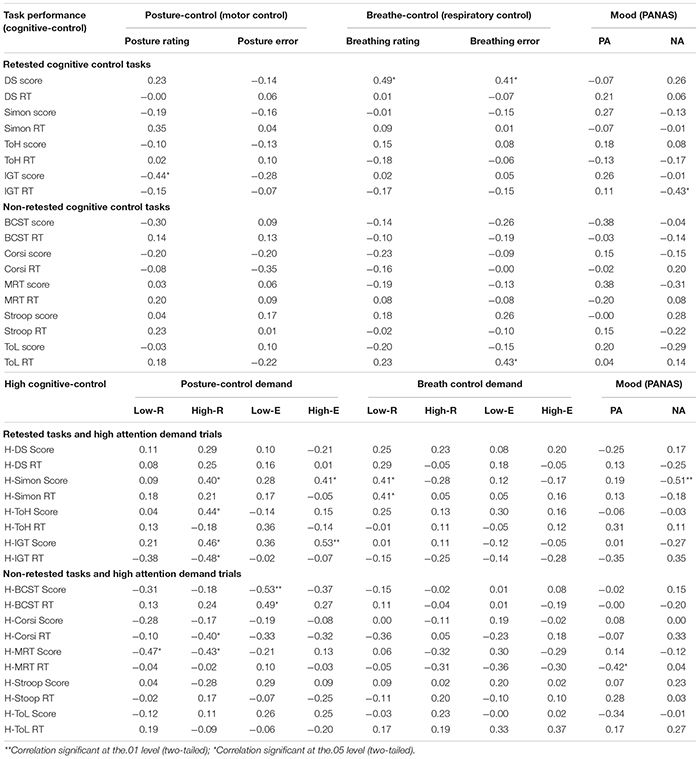

For the repeated tasks, verbal working memory (digit span) improved with changes in breath control (*r* = 0.49; *p* = 0.02). Inhibitory control in IGT accuracy diminished with improved posture control (*r* = −0.44; *p* = 0.03); however, factoring in attentional demands suggested that learning the highly challenging postures was associated with better performance on the high demanding inhibitory control (Simon task and IGT score) and planning (ToH) tasks as well as faster RTs on the IGT (all *p* < 0.05). Inattention/errors made during the high demanding postures were positively associated with improvements in inhibitory control (Simon task and IGT). Changes in regard to the less demanding breath control exercises were related to improved but slower inhibitory control (Simon accuracy and RTs).

As for the non-repeated tasks, breath control (errors) was associated with RTs for the planning tasks (ToL) (*r* = 0.43; *p* = 0.04). Attention attenuated the link between the control systems: errors with the low demand postures were associated with diminished and slower cognitive flexibility (Berg’s card sorting task accuracy and RTs). Learning the high demanding postures was associated with poorer spatial working memory (MRT) but faster RTs (Corsi RT); learning the less demanding postures was associated with worse performance on the spatial working memory task (MRT). As expected, results from both the repeated and non-repeated tasks are suggestive of attention demands altering the link between improvement in the asanas and pranayama practice and the executive control tasks. The repeated/retested tasks revealed more significant correlations as compared to the non-repeated tasks, suggesting that practice effects should be considered while studying cognitive enhancement in yoga and breath control training.

## Study 2

Cognitive functions seemed to be differentially responsive to learning the postural and breath control components, and practice effects may have confounded yoga practice-induced cognitive enhancement in Study 1 because repeated rather than ones that were novel showed more links to the training components. Attentional demands altered the association between postures, breathing, and cognitive control because yoga learning was associated with more of the cognitive tasks when attentional demands were factored in (e.g., inhibitory control), suggesting a critical role of attention in cognitive benefits. Even though the use of repeated and non-repeated tasks enabled the identification of practice effects, a non-yoga control group enables us to understand how yoga practice might facilitate executive control enhancement.

Therefore, in Study 2, we examined three components of executive control between individuals receiving 8 weeks of motor and respiratory training and a control group who did not receive yoga training. We employed the same cognitive control tasks (repeated and non-repeated), comparing performance as a function of attentional demands, maintaining the same 8-week time interval between testing.

### Materials and Methods

#### Measures

The same tasks and measures were used from Study 1: working memory tasks, planning and cognitive flexibility tasks, inhibition tasks, mood measure, and Body Mass Index (BMI) measure.

#### Participants

Twenty-seven age, gender, and education matched healthy young adults (mean age: 23.33, *SD*: 3.11; 18 men) were recruited for the study to compare against the yoga group from Study 1 (*N* = 54; control = 27). A female RA (same as in Study 1) requested participation in the control group (requesting those who have not learned yoga and are not committed to learning yoga/mindfulness practice during the period of the study), and thirty-eight participants responded to this request. Eleven participants were excluded from the control group, of which, eight participants gave baseline test but did not come for biweekly mood measures, and three participants did not come for retest. Inclusion criteria were as follows: >18 years and 8-week long non-involvement with any yoga training. The MINI was used to screen for psychiatric illnesses. All participants were non-yoga learners (non-trained and non-practicing; see Table 8 for sample descriptions). The ethics committee of the institute approved testing this control group. All participants provided signed informed consent and received a cash reimbursement (INR 500).

**TABLE 8:** Sample characteristics of the control group (*n* = 27).

**Characteristic**	**Mean (*SD*), percentage**
Age	23.33 (3.11)
Body Mass Index	20.86 (1.86)
Sex	Male: 66.67%; Female: 33.34%
Handedness	RH: 100%; LH: 0%
Vision	Corrected: 62%
Education	UG: 48.15%; PG: 51.86%

#### Procedure

After obtaining informed consent and demographic information, the same procedure as Study 1 was repeated except for the yoga training. Four cognitive tasks were administered at the start of the study (task order: digit span task, Tower of Hanoi, Simon task, and IGT). Mood measures were collected twice a week. After 8 weeks, participants were retested on the previous four tasks (repeated tasks). After a 10-min break, participants were administered the five non-repeated tasks (task order: Corsi block test, mental rotation task, Stroop task, Tower of London, and Berg’s card sorting task).

#### Variables and Analyses

To compare task performance between the two groups (yoga and control), we employed two analyses: (a) for the repeated four tasks, a mixed model analysis of variance (ANOVA) on accuracy and RTs (general and high attention demand trials) was conducted separately. Performance at baseline and at retest was the within-subjects variable, and group (yoga *vs.* control) was the between-subjects variable; age and gender were covariates. For the mood measures, positive and negative affect scores were within-subjects variables, and group was the between-subjects variable. (b) For the non-repeated five tasks, separate ANOVAs were conducted on accuracy and RTs as the dependent variable, group as the between-subjects variable and age and gender as the covariates.

### Results and Discussion

Means and standard deviations for the speed and accuracy scores across the nine tasks are shown in Table 9.

**TABLE 9:** Descriptive table of speed and accuracy in general and highly demanding trials of the tasks representing the three components of executive control of control group (*n* = 27).

**EF component**	**EF tasks**	**Task speed (RT)**	**Task accuracy (score)**	**Highly demanding trial speed (RT)**	**Highly demanding trial accuracy (score)**
Working memory	DS	72.38	84.95	18.47	–8.97
		(22.59)	(30.92)	(40.83)	(3.42)
	Corsi	70.37	74	16.11	–3.86
		(27)	(31.88)	(14.73)	(1.41)
	MRT	364.78	94.67	42.9	–10.15
		(194.84)	(25.24)	(41.43)	(30.02)
Cognitive flexibility	ToH	212.47	24.25	107.34	13.88
		(81.51)	(17.01)	(76.34)	(16.3)
	ToL	703.88	15.93	–132.84	–5.19
		(254.83)	(5.89)	(109.18)	(2.87)
	BCST	247.71	30	46.57	60.52
		(126.14)	(17.96)	(54.21)	(17.44)
Inhibition	Simon	103.25	181.19	2.88	–1.91
		(21.45)	(13.19)	(5.87)	(2.02)
	Stroop	148.08	114.89	2.95	–3.26
		(58.5)	(46.35)	(3.11)	(9.19)
	IGT	129.51	11.6	3.55	–3.52
		(63.65)	(12.29)	(14.31)	(8.22)

#### Repeated Tasks

Performance on two of the four tasks differed significantly between the yoga and control group. There was no main effect of accuracy on the DS task, but there was a DS score × group interaction, *F*(1,50) = 5.20, *p* = 0.03, $\eta_{\text{p}}^{2}$ = 0.09, experimental group showed increased accuracy from baseline (*M* = 89.20) to retest (*M* = 113.99; control group showed stable accuracy from baseline (*M* = 84.59) to retest (*M* = 84.79). There was no main effect of accuracy on the Simon task, but there was an accuracy × group interaction, *F*(1,50) = 4.58, *p* = 0.04, $\eta_{\text{p}}^{2}$ = 0.08 (experimental group baseline mean = 118.23 and retest mean = 132.17; control group baseline mean = 134.14 and retest mean = 133.46). The Simon accuracy × age interaction was also significant *F*(1,50) = 5.62, *p* = 0.02, $\eta_{\text{p}}^{2}$ = 0.10. Though the RTs did not differ significantly between the two groups for any of the tasks, and the two groups did not vary as a function of attention demands, working memory and inhibition showed performance enhancement in the yoga group.

#### Non-repeated Tasks

Results revealed significant group differences for the Stroop task. Here, accuracy was significantly higher for the experimental group, *F*(1,50) = 4.10, *p* = 0.05, $\eta_{\text{p}}^{2}$ = 0.08 (experimental group mean accuracy = 133.94 *vs.* control group = 115.36) with RTs being significantly faster for the experimental group, *F*(1,50) = 5.48, *p* = 0.02, $\eta_{\text{p}}^{2}$ = 0.10 (experimental group = 119.53 *vs.* control group = 147.44).

Independent of group, gender had an effect on RTs for the spatial working memory task (MRT), *F*(1,50) = 5.78, *p* = 0.02, $\eta_{\text{p}}^{2}$ = 0.10 such that women had slower RTs compared to men. Gender also had an effect on cognitive flexibility (Berg’s card sorting task) such that the percentage of preservative errors was greater among women compared with men, *F*(1,50) = 6.06, *p* = 0.02, $\eta_{\text{p}}^{2}$ = 0.12. The effect of age was significant for working memory such that younger compared to older participants (median 22 years) had better accuracy. For example younger participants showed higher general accuracy in Corsi block *F*(1,50) = 7.54, *p* = 0.01, $\eta_{\text{p}}^{2}$ = 0.13, and higher accuracy was observed on the demanding trials in MRT task, *F*(1,50) = 5.66, *p* = 0.02, $\eta_{\text{p}}^{2}$ = 0.10.

The two groups did not differ in terms of mood during the 8-week period. Those in the yoga group had better working memory (DS) compared to those in the control group; however, these benefits were observed for the repeated working memory task. The yoga group showed greater improvements in inhibition on the repeated (Simon task) and the non-repeated task (Stroop task) suggesting cognitive benefits might not be due to practice or task familiarity. Even though Study 1 results showed a weak link between inhibition and yoga training performance, the inhibition component of executive control might stand to benefit the most from yoga training as compared to working memory and cognitive flexibility.

## General Discussion

The present study first explored how two specific components of yoga practice, namely attention to postural control (motor control) and breath control (respiratory control), might be associated with two attributes (i.e., speed and accuracy) of executive control (working memory, planning and cognitive flexibility, and inhibition). Furthermore, the present study explored whether the relationship between motor, respiratory, and executive control alters as a function of attentional demands placed on the three control systems. We observed that attention to postural control during yoga asanas—and attention to breath control during pranayama—might be a potential mechanism through which yoga enhances specific components of executive control. Here, we report that attention to yoga postures and pranayama breathing might have revealed selective associations on the speed–accuracy tradeoff (except for cognitive flexibility assessed with the Berg’s card sorting task). This tentative assertion is in line with the unity–diversity model of executive control (Friedman and Miyake, 2017), responsiveness within specific components of executive control to specific components of yoga training might highlight the diversified nature of executive control. Further, regulation of the speed–accuracy tradeoff within the three components of executive control might be the unifying mechanism through which yoga training is influential. Studies examining the effect of mindfulness-related practice on cognitive task performance report either speed (RT) or accuracy, but not both, as in the case with working memory tasks (e.g., Jella and Shannahoff-Khalsa, 1993; Jyothsna and Rao, 2014; Sharma et al., 2014; Johnson et al., 2015; Jansen et al., 2017; Purohit and Pradhan, 2017; Crivelli et al., 2018), planning and cognitive flexibility (e.g., Levine et al., 2011; Kiani et al., 2016), and inhibitory control (e.g., Lakey et al., 2007; Semple, 2010; Alfonso et al., 2011; Moore et al., 2012; Kiani et al., 2016; Wimmer et al., 2016). Results suggest that analyzing speed–accuracy tradeoff might be useful in exploring the unifying mechanism by which yoga and other mindfulness practices might enhance executive control. We also probed and found preliminary support for yoga postures and breath-regulation exercises along with the cognitive tasks employed being linked by attentional demands placed on motor (yoga postures), respiratory (pranayama breathing), and executive resources. Further, by comparing executive control performance on repeated and non-repeated tasks, as well as performance between a yoga and control group, we attempted to address practice and ceiling effects on postintervention executive control enhancement—results revealed that task exposure (repetition) might play a critical role in pre–post comparison of cognitive enhancement, especially for working memory. Results also are indicative of inhibition component of executive control being a challenging domain in terms of being directly linked to yoga training; at the same time, it could also possibly be a domain that is most benefited by yoga training.

## Limitations and Future Directions

The nature of this investigation was exploratory; it aimed at exploring attention as a mechanism through which postural and breath control might exert domain-specific effects on cognitive control. The findings should be interpreted within the limitations of our approach. For instance, cognitive enhancement due to physical exercise has been analyzed using sample sizes smaller than those utilized in the present study (e.g., McMorris and Graydon, 1997; Kruk et al., 2001; Draper et al., 2010); nevertheless, sample size in Study 1 is a limitation. To understand the extent of these limitations, we carried out retrospective power analysis (using G^∗^power) and observed that the correlation values obtained in Study 1 were within the critical range, but the beta errors exceeded the acceptable limit (Banerjee et al., 2009). Some researchers observe that the retrospective power analysis violates the key assumptions of a random sample (Zhang et al., 2019); however, the results of Study 1 should be interpreted within the limitation of small sample size and its impact on the power to detect significant correlations. Similarly, the non-random assignment of participants to the two groups also limits generalization; however, the yoga group represents a population that self-selects and seeks yoga training for cognitive benefits. Others have also noted that the choice of a control group in mindfulness-based research poses a great challenge (Kinser and Robins, 2013). To verify the extent of this limitation, we carried out a retrospective power analysis for Study 2 (using G^∗^Power), and the results suggested that the obtained *F* values were well within the critical range, and the beta error were also within the acceptable limit. The lack of an intelligence test was also a limitation; however, participants were students from an educational institute and were admitted through a highly competitive national level entrance test. Yoga asanas were followed by pranayama; counter-balancing the order of yoga postures and pranayama breathing was not possible. Thus, order effects regarding the training must be considered. While we ensured that only yoga-naïve participants participated and practiced only in the presence of the instructor (no home-based practice), we did not control for other activities that might have influenced physical and respiratory fitness during the 8-week study intervals. Finally, testing of the yoga and control group took place at two different points (due to budget/resource constraints, specifically participant payment).

## Conclusion

Specific components of the speed–accuracy tradeoff in regard to cognitive control performance might have been differentially responsive to specific aspects of yoga training within this young adult sample. Yoga training is commonly imparted as a combination of postures and breath exercises and occasionally is combined with other activities such as listening to music, chanting, deity worship, experience-sharing, and motivational speeches (e.g., Manjunath and Telles, 2001; Levine et al., 2011; Kiani et al., 2016). Each of these activities could differentially influence executive control. Conversely, there could be a possibility that different components of executive control could respond uniquely to yoga training. Precise effects of multiple components that form a yoga practice, and the differential effects of each component on specific domains of executive control, will help address issues regarding the lack of a definition, mechanism, and established causality on the enhancement of executive control through yoga training. The present findings, though exploratory, provide preliminary support to this endeavor.

## Data Availability Statement

The raw data reported in this article are available from the corresponding author upon request.

## Ethics Statement

The studies involving human participants were reviewed and approved by Institute Ethics Committee (IEC No. P003), Indian Institute of Technology, Delhi. The patients/participants provided their written informed consent to participate in this study.

## Author Contributions

VS: conceptualization, funding acquisition, investigation, methodology, analyses, and original draft preparation. VM: data entry and coding, research assistance, and project administration.

## Conflict of Interest

The authors declare that the research was conducted in the absence of any commercial or financial relationships that could be construed as a potential conflict of interest.

## Appendix 1

### Observation Sheet

Rater’s name: ___________________________________________________________

Session no. ____________ Date: __________________Time: ______________________

Participants:

### Section I: Postures

Please follow these rating instructions

(A) Rate each posture, posture-attaining process using the following scale to indicate how well the posture was performed. The Instructor’s posture will be used as an ideal comparison for the ratings.

**TABLE A1:** Observation sheet for posture ratings and posture errors.

**Posture**	**Duration**	**Attributes**	**P1**	**P2**	**P3**	**P4**	**P5**
Padahastasana		Rating					
		Error Freq.					
Virabhadrasana*		Rating					
		Error Freq.					
Trikonasana*		Rating					
		Error Freq.					
Katichakrasana		Rating					
		Error Freq.					
Tadasana		Rating					
		Error Freq.					
Ardha Chakrasana		Rating					
		Error Freq.					
Pranamasana*		Rating					
		Error Freq.					
Vrikasana*		Rating					
		Error Freq.					

**Bilaterally done postures (1 min per side).*

### Section II: Breathing

(A) Rate each breathing exercise and breathe-exercise process using the following scale to indicate how well the breathing exercises were performed. The Instructor’s breathing session will be used as an ideal comparison for the ratings.

**TABLE A2:** Observation table for breathing exercise, and breathing exercise errors.

**Breathing**	**Duration**	**Attributes**	**P1**	**P2**	**P3**	**P4**	**P5**
Abdominal breathing	5 min	Rating					
		Error Freq.					
Thoracic breathing		Rating					
		Error Freq.					
Brahma mudra		Rating					
		Error Freq.					
Alternate nostril breathing		Rating					
		Error Freq.					
Observing breath		Rating					
		Error Freq.					
							
							
							
							
							
Special note/Observations:							
Rater’s signature and date:							
